# Comparison of denaturing agent effects in enzymatic *N*-glycan release for human plasma *N*-glycan analysis

**DOI:** 10.55730/1300-0527.3457

**Published:** 2022-05-20

**Authors:** H. Mehmet KAYILI, Rokia SAKHTA, Bekir SALİH

**Affiliations:** 1 Department of Biomedical Engineering, Faculty of Engineering, Karabük University, Karabük, Turkey; 2 Department of Chemistry, Faculty of Science, Hacettepe University, Ankara, Turkey

**Keywords:** Glycomics, glycan release, denaturing agents, procainamide labeling, human plasma, detergents

## Abstract

Glycosylation is an essential posttranslational modification observed in the living proteome. Glycosylation profiles in glycoproteins can change in commonly observed diseases such as cancer. Identifying these changes is crucial in discovering new biomarkers for the early diagnosis of cancer. One of the main steps of *N*-glycan analysis is to release *N*-glycans from glycoproteins by specific enzymes. The study compares common denaturing agent combinations used in *N*-glycan release methods. In the study, human plasma was used to test the release methods of *N*-glycans containing different detergent combinations. The released *N*-glycans were labeled with the procainamide tag, purified using cellulose-containing solid-phase extraction cartridges, and analyzed by high-performance liquid chromatography-hydrophilic interaction liquid chromatography equipped with fluorescence detection (HPLC-HILIC-FLD). The results showed that the sodium dodecyl sulfate and sodium deoxycholate (SDS + SDC) detergent combination provided the highest average FLD signal areas and intensities in the *N*-glycan analysis. The protocol with SDS resulted in more reproducible average FLD signal areas and intensities. It was also found that the average signal FLD signal areas and intensities of the detected *N*-glycans were reduced when SDS and SDC were used with 1,4-dithiothreitol (DTT) reducing agents. In addition, deglycosylation of glycoproteins with various denaturing agents resulted in relatively minor variation in human plasma *N*-glycosylation profiles.

## 1. Introduction

*N-*linked glycosylation is one of the most defined posttranslational modifications in nature [1]. Glycosylation regulates the critical functions of proteins and plays a vital role in healthy and diseased conditions [2]. *N-*glycans have the potential to be used in the early diagnosis of some cancers [3]. It is also known that most of the approved biomarkers are glycoproteins or glycoconjugates [4, 5]. Therefore, *N-*glycan analysis of glycoproteins derived from biological samples is crucial for discovering new biomarker candidates.

Glycomics is the field that focuses on elucidating glycan structures and quantification of glycans [6]. Clinical application of quantitative glycomics has attracted attention because aberrant glycosylation is associated with many diseases [7–10]. The *N-*glycan release is one of the most critical steps for quantitative glycomics [11]. *N-*glycans are released from glycoproteins by an enzyme namely PNGase F (*N*-glycosidase F). Glycoproteins are first denatured by detergents prior to enzymatic treatments to facilitate access of glycans to the PNGase F. It has been examined that many parameters in *N-*glycan release methods influence the profiles of *N-*glycans derived from glycoproteins [12]. For example, the kinetics of *N-*glycan release by PNGase F enzyme resulted in different IgG glycosylation profiles [13]. On the other hand, different *N-*glycan release protocols were examined for cell line analysis and, in-solution approach has been found to be the most robust [14]. In addition, a study has showed that different PNGase F enzymes resulted in diverse IgG and human plasma *N-*glycosylation profiles [12]. It is clearly seen that the studies for optimizing of *N-*glycan release methods are required to improve the clinical glycomics applications.

The first step in sample preparation for glycomic experiments includes the extraction of proteins from biological samples. Since most of the glycoproteins are integral membrane proteins, the use of detergents is obligatory for maximizing the extraction of proteins [15]. Many detergents have been introduced and compared regarding the efficient protein extraction for mass spectrometry-based proteomics [16, 17]. These detergents can also be used for quantitative glycomic experiments. However, the effects of commonly used detergents on the enzymatical *N-*glycan release process have not been examined yet.

In the study, different denaturing agents and their combinations were investigated for quantitative glycomics performed by high-performance liquid chromatography-hydrophilic interaction liquid chromatography equipped with fluorescence detection (HPLC-HILIC-FLD). The *N-*glycans were released from human plasma glycoproteins with different detergents and detergent combinations, labeled with procainamide tag, and purified by cellulose-containing solid-phase extraction cartridges. The obtained data were evaluated based on the total FLD signal areas, intensities, and relative abundances of the detected *N-*glycan peaks.

## 2. Materials and methods

Human plasma, cellulose, Igepal Ca-630, sodium dodecyl sulfate (SDS), sodium deoxycholate (SDC), 1,4-Dithiothreitol (DTT), phosphate buffer saline (PBS), dimethyl sulfoxide (DMSO), deionized water (dH_2_O) for HPLC-HILIC-FLD analysis, acetonitrile (ACN), trifluoroacetic acid (TFA), and sodium cyanoborohydride were supplied from sigma Aldrich: (St Louis, MO, USA). Acetic acid (LC/MS grade, AA) was purchased from Carlo Erba Reagents. PNGase F enzyme was obtained from Promega (Madison, WI, USA). Procainamide HCl was purchased from Abcam (Cambridge, UK). Deionized water (dH_2_O) was obtained using an Expe-Ultrapure Water System (Mirae St., Korea) for using in sample preparation steps.

### 2.1. Glycan release of human plasma glycoproteins with different detergents

The protocol was applied by following our previously described method [18]. Firstly, human plasma was prepared with a concentration of 70 μg μL^−1^ using its lyophilized form. The following detergents were prepared: 2% SDS (w/v) (**1**), 2% SDC (w/v) (**2**), 2% SDS (w/v) including 0.5 M DTT (**3**), 2% SDC (w/v) including 0.5 M DTT (**4**), 2% SDS + 2% SDC mixture (v/v, 1/1) (**5**), and 2% SDS+ 2% SDC mixture (v/v) including 0.5M DTT (**6**). Then, 20 μL of human plasma was mixed with 20 μL of each detergent. Subsequently, the samples were incubated at 60 °C for 10 min to denature the glycoproteins. Twenty microliters of 4% Igepal-CA630 and 20 μL of 5X PBS were added to the samples, respectively. Finally, 1 U of PNGase F enzyme was inserted into the samples and all samples were incubated at 37 °C for 16 h.

### 2.2. Procainamide labeling of released *N*-glycans

The glycan release samples were mixed with 100 μL of a labeling solution, including 50 μL of procainamide hydrochloric acid (110 mg mL^−1^ in DMSO/Acetic Acid, 7/3, v/v) and 50 μL of sodium cyanoborohydride (65 mg mL^−1^ in DMSO/AA, 7/3, v/v). Then, the samples were incubated at 65 °C for 2 h.

### 2.3. Purification of procainamide labeled *N*-glycans

Purification of procainamide labeled *N*-glycans was achieved by using SPE cartridges. A solution of microcrystalline cellulose (100 mg mL^−1^) in dH_2_O was freshly prepared and a 300 μL microcrystalline cellulose solution was inserted into the microcentrifuge tubes. The microcrystalline cellulose-containing SPE cartridges were washed with 1 mL of dH_2_O and 1 mL of ACN/dH_2_O, 85/15, v/v for three times, respectively. Then, the glycan release samples (120 μL) were mixed with 680 μL of ACN to obtain proper loading conditions (85/15, v/v, ACN/sample). The microcrystalline cellulose was mixed with the loading samples, and the samples were incubated at room temperature in a thermomixer by shaking at 500 rpm for 15 min. Then, the slurry was transferred to SPE cartridges, and the loading samples were discarded by applying a vacuum. The microcrystalline cellulose-containing SPE cartridges were washed with 1 mL of ACN/dH_2_O/TFA mixture (85/14/1, v/v/v) and 1 mL of ACN/dH_2_O mixture (85/15, v/v) three times, respectively. The procainamide labeled *N*-glycans were eluted with 0.75 mL of water. The eluates were dried with a speed vacuum concentrator overnight. The dried samples were dissolved in a mixture of 100 μL of ACN/dH_2_O, 75/25, v/v, and transferred to the vials for HPLC-HILIC-FLD analysis.

### 2.4. HPLC-HILIC-FLD analysis

An Agilent 1200 series HPLC system with Agilent 1260 FLD detector was employed for procainamide labeled *N*-glycan analysis. The analysis was achieved using a Waters Glycan BEH Amide 2.5 μm (2.1 mm ID × 15 cm L) column. The wavelengths of the FLD detector for excitation and emission were set to 310 and 370, respectively. 100% ACN and 50 mM ammonium formate pH:4.4 were used as mobile phase A and mobile phase B, respectively. Mobile phase A was set from 75% to 53% in 60 min for the analytical separations. The flow rate was 0.25 mL min^−1^. The injection volume was set to 10 μL.

### 2.5. Identification of procainamide labeled *N*-glycans

Identification of *N-*glycan peaks of human plasma in the FLD chromatogram was achieved by HPLC-HILIC-FLD-MS/MS analysis previously with our group [19]. The area and intensity values of procainamide labeling *N*-glycans were conducted using the OpenLAb software (Agilent Technologies, Santa Clara, USA). The total FLD signal area or intensity values were calculated by summing the areas of the detected *N*-glycans for each denaturing agent-containing method. In addition, the detected areas and intensities of each *N*-glycans were averaged for use in comparisons. The relative intensities and areas of each detected *N-*glycans were calculated by applying the total area normalization approach.

## 3. Results and discussion

In this study, the human plasma *N*-glycans were released using the methods including various detergent combinations, labeled by the procainamide tag, and purified via cellulose-containing solid-phase extraction cartridges. Then, *N*-glycan analysis was performed by an HPLC-HILIC-FLD. The detected *N*-glycan peak areas and intensities were processed by software and subsequently evaluated. The experiments were achieved on two different days with three replicates (n = 6). In the study, we compared the six detergent combinations commonly used in the *N*-glycan release methods as shown in Table. Since most manufacturers of PNGase F offer SDS and DTT as denaturing agents, and the use of SDC in glycoproteomics applications has increased, these denaturing agents and their combinations were selected in the study for comparison.

**Figure 1:**
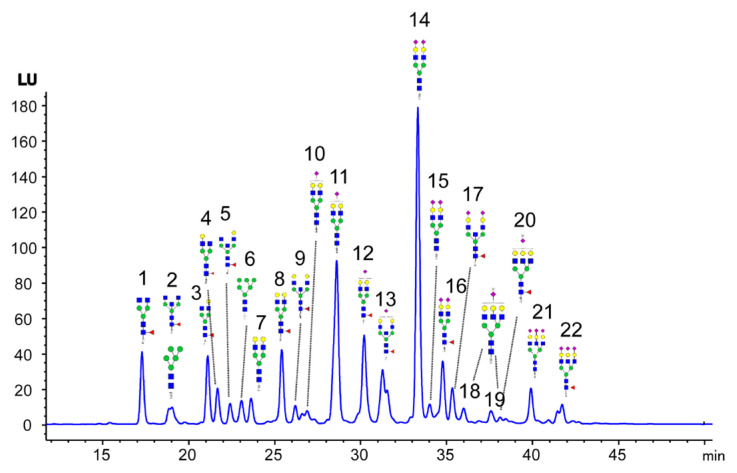
A typical chromatogram of procainamide labeled *N*-glycans of human plasma profiled by HPLC-HILIC-FLD.

Figure 1 presents an example FLD chromatogram obtained from the analysis of the procainamide labeled *N*-glycans by the HPLC-HILIC-FLD. We detected 22 *N*-glycan peaks belonging to human plasma *N*-glycome in this study. The peaks were annotated based on the literature knowledge and our previous work (18, 19). Then, the obtained data were compared among *N*-glycan release methods with detergent combinations.

### 3.1. Comparison of detergent combinations in *N*-glycan release methods based on peak areas and peak intensities

**Figure 2:**
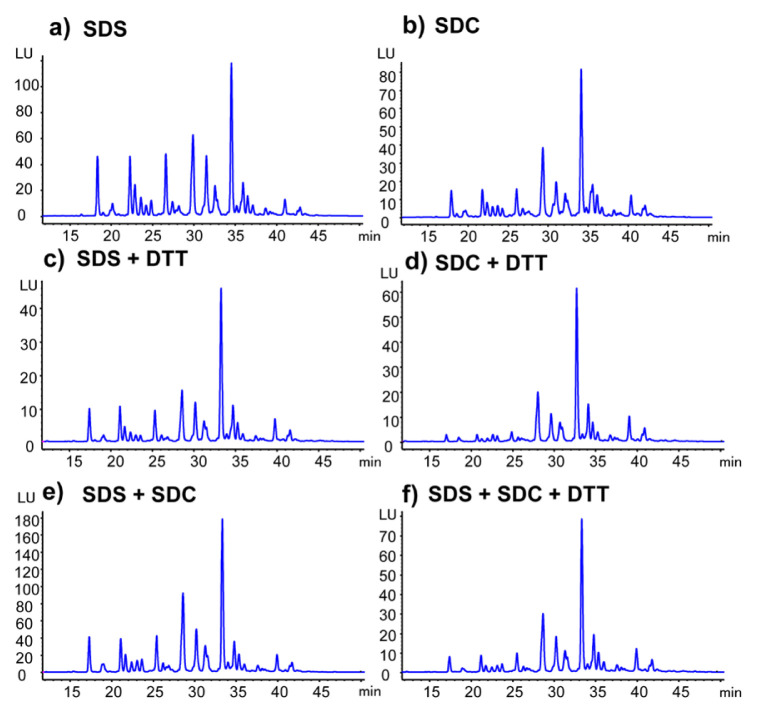
FLD chromatograms of human plasma *N*-glycans obtained from different detergent combination containing *N*-glycan release methods (a) 2% SDS, (b) 2% SDC, (c) 2% SDS + 0.5 M DTT, (d) 2% SDC + 0.5 M DTT, (e) 2% SDS + 2% SDC, (f) 2% SDS + 2% SDC + 0.5 M DTT.

The detected peaks belonging to *N*-glycans of human plasma were found in each FLD chromatogram obtained from *N*-glycan release methods containing different detergent combinations (Figure 2). The resolution and shape of the *N*-glycan peaks were not influenced by the *N*-glycan release methods with different detergent combinations.

**Figure 3:**
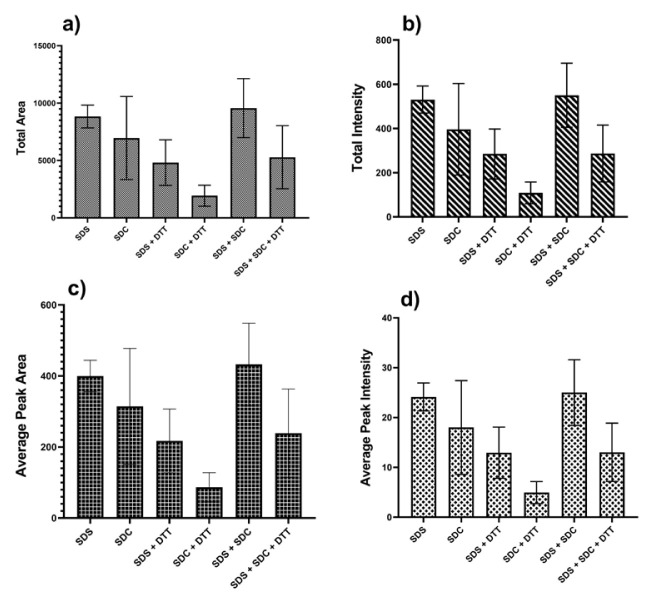
Total and average peak areas of the *N*-glycan peaks obtained from *N*-glycan release methods including different detergent combinations.

The *N*-glycan release methods with different detergent combinations were first compared by evaluating the peak areas of *N*-glycans extracted from the FLD chromatograms. Figure 3a shows the comparison of data based on total peak areas of the detected *N*-glycan peaks. The highest peak areas were calculated for each detergent containing *N*-glycan release methods in the FLD chromatogram. The SDS + SDC detergent combination used in the *N*-glycan release methods was found to have the highest total peak area. The *N*-glycan release approach that used SDS was found to have the second-highest area. The *N*-glycan release methods with different detergents were also compared by evaluating the peak intensity of *N*-glycans extracted from the FLD chromatogram. Figure 3b displays the comparison of the data based on peak intensities of the detected *N*-glycan peaks. As expected, the SDS + SDC detergent combination was the highest total peak intensities in the FLD chromatogram when the obtained intensity values were compared in the *N*-glycan release methods. The SDS detergent containing the *N*-glycan release method was found to have the second-highest intensity. The data was ordered based on total areas and intensities as follows: SDS + SDC > SDS > SDC > SDS + SDC + 0.5 M DTT > SDS + DTT > SDC + 0.5 M DTT. On the other hand, the average areas and intensities obtained from each method were evaluated to test the efficiency of *N*-glycan analysis. The results obtained from the total area and intensity values of the *N*-glycans were matched with the results from average areas and intensities (Figures 3c and 3d). The *N*-glycan release method with SDS detergent provided more reproducibility based on total and average peak areas and intensities.

### 3.2. Comparison of detergent combinations in *N*-glycan release methods based on relative abundances

**Figure 4:**
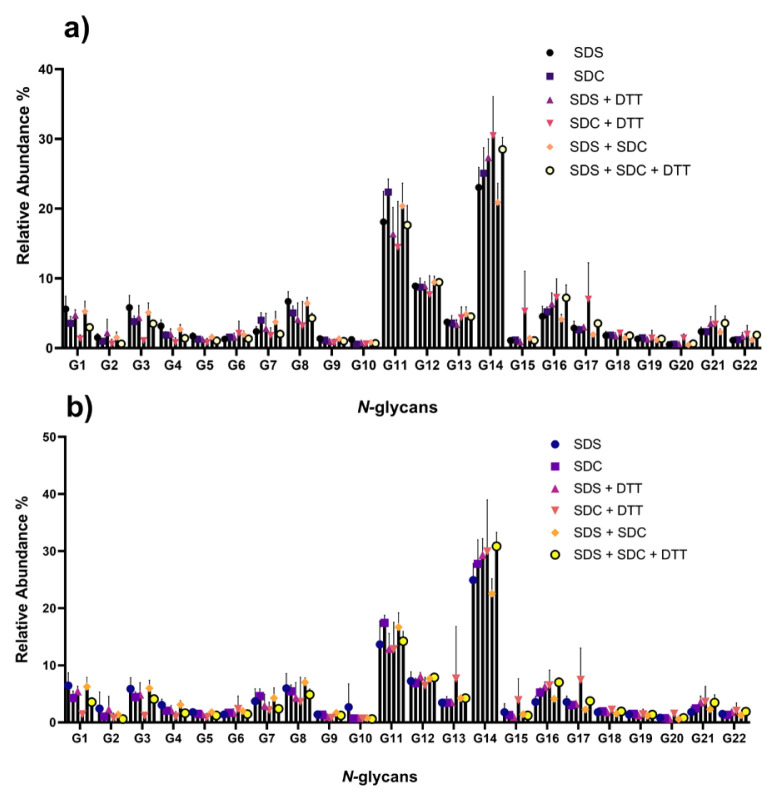
Relative abundances of the detected *N*-glycans calculated using (a) peak areas and (b) peak intensities.

The total area normalization approach was used for the calculation of the relative abundances of the *N*-glycans. The calculated relative abundances of each *N*-glycan based on peak areas and intensities are shown in Figures 4a and 4b, respectively. The relative abundances of the detected peaks are also shown in Tables S1 and S2 based on peak areas and peak intensities, respectively. The data resulted in relatively minor changes in relative abundances of human plasma *N*-glycans among *N*-glycan release methods, including different detergent combinations. However, the *N*-glycan G14 (H5N4S2, di-antennary sialylated type) abundantly found in human plasma differed among protocols. It had a higher abundance in DTT-containing protocols (Tables S1 and S2). In addition, the relative abundances of tri-antennary sialylated species were determined higher in DTT reducing agent-containing methods than other methods worked in this study. For human plasma glycoproteins, the protein structures in their native form may form steric hindrance, which restricts the access of any PNGase F enzyme to defined glycosylation sites. Methods involving reducing agents can remove the steric hindrance found in proteins and allow the PNGase F enzyme to more efficiently reach the glycosylation sites.

The area-based CV% (coefficient of variation) of the peak abundances was also investigated for *N*-glycan release methods with different detergent combinations (Figure S1). It was found that SDS + DTT and SDC + DTT containing *N*-glycan release methods had higher mean CV% (it was 32% and 63% for SDS + DTT and SDC + DTT, respectively). However, the mean CV% values of plasma *N*-glycan abundances for SDS, SDC, SDS + SDC and, SDS + SDC + DTT detergent containing *N*-glycan release method were detected to be relatively low. The average CV% of these *N*-glycan release methods was 26%, 18%, 24%, and 20%, respectively.

The main limitation of this study was to compare three different detergents and their combinations, whereas many others are available in *N-*glycan release methods. Many manufacturers producing the PNGase F enzyme offer SDS-based *N-*glycan release methods. These methods were applied with DTT reducing agents. Therefore, the most widely used chemicals for that purpose were evaluated in the study. On the other hand, SDC has been recently evaluated in releasing *N*-glycans from glycoproteins extracted from biological samples [20]. They have determined that SDC assisted approach was found to be more efficient compared with filter-aided sample preparation. On the other hand, the purification of procainamide labeled *N-*glycans may also affect the data obtained from *N-*glycan analysis. In the literature, many purification methods, including different interactions between *N-*glycans and adsorbents, have been introduced [21, 22]. For HILIC-FLD-based *N-*glycosylation analysis, hydrophilic-interaction-based sorbents have commonly been employed for the purifications [23]. It has been detected that HILIC-based sorbents showed good reproducibility [24]. Therefore, we used the self-packed cellulose solid-phase extraction cartridges for that purpose in this study.

In our study, the *N-*glycan profiles of human plasma were found to have minor changes in different detergent combinations containing *N-*glycan release methods. It could be concluded that the denaturation agents influence the efficiency of *N-*glycan release. In addition, the removal of *N-*glycan types was found to differ based on *N-*glycan release methods with different detergent combinations. A recent study evaluated the PNGase F enzymes produced by three manufacturers regarding the *N-*glycan profiles. They have found that deglycosylation with PNGase F enzymes manufactured by different companies resulted in different IgG and plasma *N-*glycosylation HILIC-FLD profiles [12]. These results indicated that the applied *N-*glycan release method, including denaturing agents and PNGase F enzymes, provided different *N-*glycosylation HILIC-FLD profiles.

## Supporting Information

**Table S1.** Relative abundances of *N-*glycan peaks based on total area.

	SDS		SDC		SDS + DTT		SDC + DTT		SDS + SDC		SDS + SDC + DTT	
	Mean	SD	Mean	SD	Mean	SD	Mean	SD	Mean	SD	Mean	SD
G1	5.59	1.87	3.55	0.99	4.72	0.77	1.32	0.60	5.26	1.46	2.97	0.35
G2	1.52	0.56	0.99	0.26	2.17	1.96	0.60	0.68	1.54	0.76	0.62	0.53
G3	5.82	1.71	3.82	0.80	4.40	1.72	1.08	0.40	5.10	1.38	3.49	0.33
G4	3.16	0.89	1.86	0.48	1.99	0.79	0.79	0.70	2.70	0.68	1.41	0.15
G5	1.70	0.50	1.29	0.26	1.26	0.36	0.78	0.62	1.59	0.34	1.06	0.10
G6	1.28	0.17	1.55	0.30	1.56	0.57	2.12	1.75	1.92	0.53	1.34	0.16
G7	2.37	0.71	3.99	1.09	2.81	2.17	1.81	1.10	3.75	1.51	2.01	0.58
G8	6.69	1.38	5.03	1.01	4.11	2.37	3.19	3.47	6.41	0.86	4.29	0.71
G9	1.34	0.24	1.14	0.32	0.97	0.39	0.77	0.52	1.34	0.27	1.00	0.28
G10	1.22	0.15	0.57	0.09	0.79	0.25	0.59	0.45	0.82	0.13	0.68	0.29
G11	18.09	4.38	22.36	1.87	16.37	3.81	14.46	6.54	20.39	3.26	17.62	2.81
G12	8.89	0.44	8.73	1.30	8.91	0.61	7.64	2.73	9.44	0.90	9.43	0.57
G13	3.73	0.36	3.58	1.09	3.39	0.67	4.35	1.53	4.84	1.08	4.52	0.18
G14	23.04	2.89	25.07	3.68	27.33	2.67	30.45	5.62	20.88	2.74	28.50	1.72
G15	1.10	0.34	1.16	0.16	0.93	0.39	5.28	5.75	1.42	0.34	1.10	0.39
G16	4.53	1.48	5.21	0.66	6.34	1.58	7.27	2.67	4.12	0.73	7.21	1.85
G17	2.87	0.99	2.62	0.51	3.03	0.30	7.01	5.22	1.95	0.29	3.54	0.21
G18	1.78	0.63	1.85	0.22	1.75	0.45	2.17	0.26	1.44	0.59	1.78	0.20
G19	1.30	0.46	1.47	0.18	1.31	0.24	1.44	1.12	1.16	0.39	1.33	0.13
G20	0.50	0.22	0.58	0.07	0.62	0.06	1.44	0.59	0.46	0.19	0.64	0.10
G21	2.36	0.65	2.37	0.26	3.55	0.96	3.44	2.63	2.29	0.48	3.57	1.05

**Table S2.** Relative abundances of *N-*glycan peaks based on total intensity.

	SDS		SDC		SDS + DTT		SDC + DTT		SDS + SDC		SDS + SDC + DTT	
	Mean	SD	Mean	SD	Mean	SD	Mean	SD	Mean	SD	Mean	SD
G1	6.44	2.24	4.27	1.22	5.40	0.92	1.39	0.58	6.25	1.64	3.55	0.45
G2	2.40	2.91	1.02	0.25	2.27	2.29	0.73	0.87	1.38	0.40	0.58	0.39
G3	5.87	1.97	4.43	0.92	4.88	2.08	1.16	0.46	5.98	1.40	4.08	0.52
G4	3.05	1.02	2.05	0.59	2.15	0.80	0.96	0.93	3.09	0.73	1.62	0.25
G5	1.78	0.58	1.49	0.29	1.41	0.41	0.88	0.71	1.78	0.34	1.20	0.15
G6	1.37	0.20	1.69	0.26	1.67	0.71	2.34	2.26	2.00	0.45	1.46	0.11
G7	3.70	2.19	4.60	1.28	2.88	2.04	2.10	1.45	4.26	1.80	2.38	0.79
G8	5.95	2.62	5.43	1.14	4.31	2.66	3.59	4.31	7.01	0.75	4.87	0.99
G9	1.38	0.28	1.34	0.44	1.02	0.33	0.77	0.31	1.67	0.29	1.21	0.30
G10	2.66	4.08	0.68	0.06	0.77	0.18	0.66	0.39	0.77	0.09	0.58	0.12
G11	13.65	4.12	17.41	1.36	12.89	2.70	12.82	4.72	16.69	2.50	14.21	1.70
G12	7.23	1.63	6.87	0.86	8.04	0.70	6.44	1.48	7.66	0.50	7.87	0.43
G13	3.46	0.61	3.42	1.10	3.55	0.44	7.69	9.11	4.25	0.36	4.25	0.19
G14	24.91	2.91	27.76	4.22	29.32	2.87	29.92	9.06	22.56	2.59	30.84	2.47
G15	1.79	1.50	1.30	0.16	0.97	0.30	3.89	3.78	1.42	0.35	1.21	0.18
G16	3.57	0.98	5.25	0.78	6.24	0.84	6.49	2.67	4.12	0.58	7.01	0.81
G17	3.57	1.02	3.03	0.52	3.27	0.23	7.44	5.55	2.27	0.32	3.74	0.29
G18	1.78	0.69	1.95	0.18	1.76	0.43	2.20	0.45	1.54	0.63	1.91	0.14
G19	1.37	0.77	1.49	0.17	1.26	0.24	1.40	0.92	1.18	0.42	1.35	0.15
G20	0.80	0.64	0.73	0.10	0.61	0.15	1.51	0.61	0.56	0.27	0.79	0.12
G21	1.80	0.93	2.45	0.23	3.51	1.10	3.60	2.70	2.30	0.43	3.45	1.38
G22	1.46	0.55	1.33	0.17	1.80	0.53	2.02	1.37	1.27	0.20	1.87	0.55

Figure S1. Comparison of six different detergent combinations used in *N-*glycan release methods. Reproducibility of human plasma *N-*glycome quantification represented by area coefficient of variation (CV%) values for 22 *N-*glycan peaks.

## Figures and Tables

**Table.** The denaturing agents used in *N*-glycan release methods.

	Detergent combinations	Initial concentration	Final concentration before PNGase F treatment
1	SDS	2%	0.5%
2	SDC	2%	0.5%
3	SDS + DTT	2% + 500 mM	0.5% + 125 mM
4	SDC + DTT	2% + 500 mM	0.5% + 125 mM
5	SDS + SDC	1% + 1%	0.25% + 0.25%
6	SDS + SDC + DTT	1% + 1% + 0.5M	0.25% + 0.25% + 0.125M
