# Supplementary figures and images for: Comparison of denaturing agent effects in enzymatic N-glycan release for human plasma N-glycan analysis
Source: Turk J Chem. 2022 May 20;46(5):1524–30. doi: 10.55730/1300-0527.3457 (PMC10390200; doi:10.55730/1300-0527.3457)

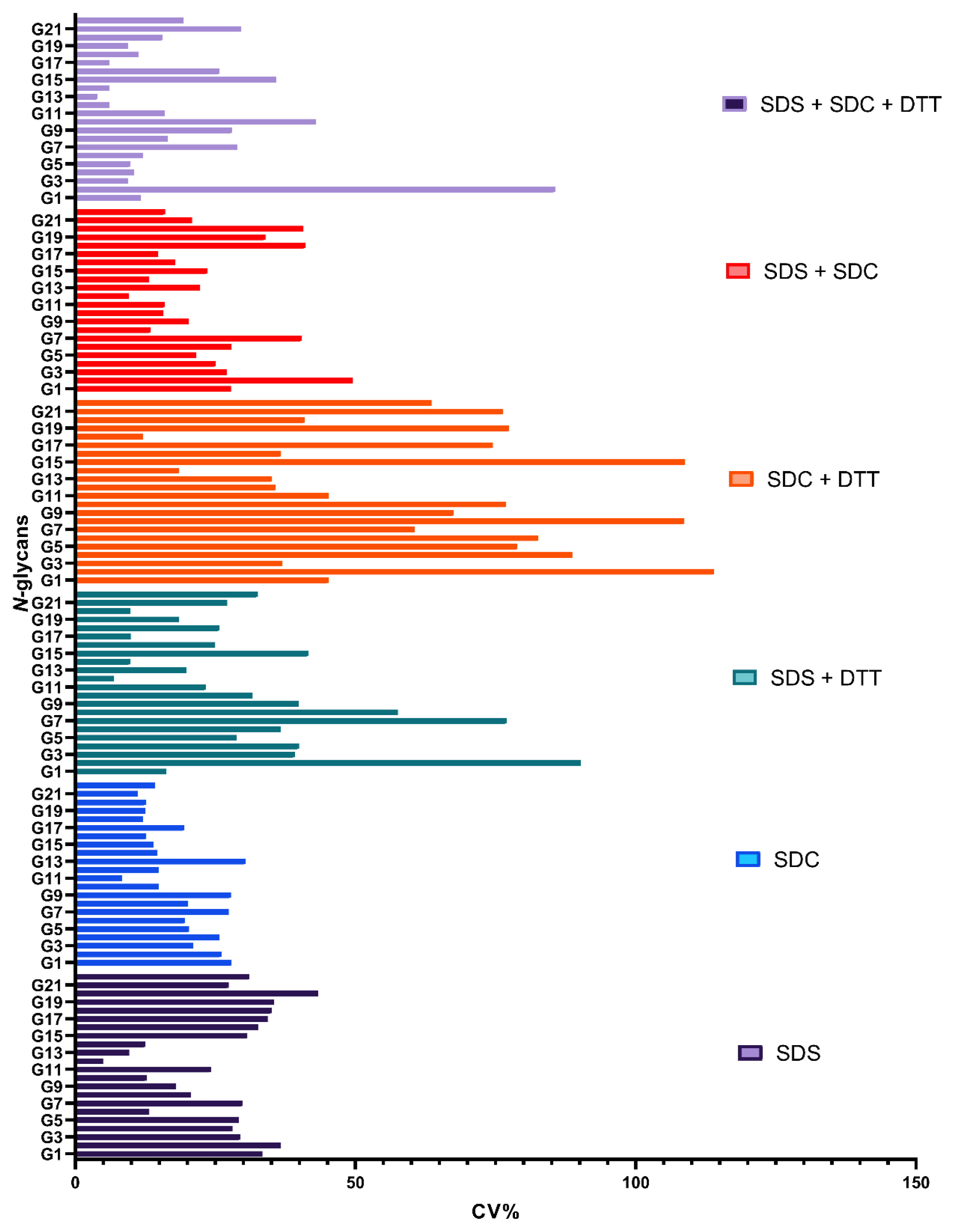

Supplement: Figure S1. — Comparison of six different detergent combinations used in N-glycan release methods. Reproducibility of human plasma N-glycome quantification represented by area coefficient of variation (CV%) values for 22 N-glycan peaks. [file turkjchem-46-5-1524s1.tif]
